# Identification of genes associated with growth cessation and bud dormancy entrance using a dormancy-incapable tree mutant

**DOI:** 10.1186/1471-2229-10-25

**Published:** 2010-02-09

**Authors:** Sergio Jiménez, Zhigang Li, Gregory L Reighard, Douglas G Bielenberg

**Affiliations:** 1 Department of Horticulture, Clemson University, Clemson, SC 29634-0319, USA; 2 Department of Biological Sciences, Clemson University, Clemson, SC 29634-0314, USA

## Abstract

**Background:**

In many tree species the perception of short days (SD) can trigger growth cessation, dormancy entrance, and the establishment of a chilling requirement for bud break. The molecular mechanisms connecting photoperiod perception, growth cessation and dormancy entrance in perennials are not clearly understood. The peach [*Prunus persica *(L.) Batsch] *evergrowing *(*evg*) mutant fails to cease growth and therefore cannot enter dormancy under SD. We used the *evg *mutant to filter gene expression associated with growth cessation after exposure to SD. Wild-type and *evg *plants were grown under controlled conditions of long days (16 h/8 h) followed by transfer to SD (8 h/16 h) for eight weeks. Apical tissues were sampled at zero, one, two, four, and eight weeks of SD and suppression subtractive hybridization was performed between genotypes at the same time points.

**Results:**

We identified 23 up-regulated genes in the wild-type with respect to the mutant during SD exposure. We used quantitative real-time PCR to verify the expression of the differentially expressed genes in wild-type tissues following the transition to SD treatment. Three general expression patterns were evident: one group of genes decreased at the time of growth cessation (after 2 weeks in SD), another that increased immediately after the SD exposure and then remained steady, and another that increased throughout SD exposure.

**Conclusions:**

The use of the dormancy-incapable mutant *evg *has allowed us to reduce the number of genes typically detected by differential display techniques for SD experiments. These genes are candidates for involvement in the signalling pathway leading from photoperiod perception to growth cessation and dormancy entrance and will be the target of future investigations.

## Background

Dormancy is defined as the inability to initiate growth from meristems under favourable conditions [1]. The first step towards establishing dormancy is growth cessation. Photoperiod has been known to govern growth cessation and dormancy entrance in many perennial species in temperate climates [2,3], including peach [*Prunus persica *(L.) Batsch]. Bud formation is concomitant with dormancy entrance, although it is not required and seems to be independent of dormancy establishment [1]. Cold acclimation may also be induced by some of the same environmental factors as bud dormancy but does not appear to be mechanistically linked to dormancy induction [4,5].

Several recent studies have used global approaches to analyze the molecular mechanisms of dormancy. Expression profiling during dormancy induction, maintenance and release were analyzed in *Populus tremula *[6], *P. tremula *× *P. alba *[5,7], *P. deltoides *Bartr. ex Marsh [4], Norway spruce [8], oak [9], leafy spurge [10-12], raspberry [13], grapevine [14], peach [15], and apricot [16]. These studies have described an initial set of candidate genes involved in cold- or light-induced dormancy in tree species.

The use of transgenic mutants for comparative analysis has been another approach to analyze the molecular mechanism of dormancy. There is evidence that the short day (SD) dormancy-inducing signal is mediated through phytochrome and the *FLOWERING TIME *(*FT*)/*CONSTANS *(*CO*) module [17,18]. *P. tremula *× *P. tremuloides *trees over-expressing *FT *do not stop growing upon exposure to SD and bud set could be induced independently from SD by down-regulation of *FT *[18]. It has also been proposed that a change in carbohydrate metabolism could induce ethylene biosynthesis before the formation of the bud structure [5,19]. Over-expression of *ABCISIC ACID-INSENSITIVE3 *(*ABI3*) gene in poplar prevents the formations of closed apical buds upon SD induction, indicating that abscisic acid (ABA) could contribute to the transition to closed bud [5,20]. However, only certain downstream components of the signal transduction chain are known and their connections are poorly characterized. The molecular mechanisms responsible for growth arrest are still not clearly understood, because of bud formation, growth cessation and cold acclimation processes overlap in time with seasonal changes in light quality, temperature, and day length. Responses to SD and low temperature conditions independent of growth cessation and dormancy-induction complicate global gene expression analyses, particularly when experiments are performed in the field during natural seasonal transitions [5]. It is therefore difficult to associate molecular changes with specific physiological events.

The peach mutant *evergrowing *(*evg*), a non-dormant genotype identified from southern Mexico, fails to cease growth and enter dormancy under dormancy-inducing conditions [21,22]. The *evg *mutant does not form apical buds in response to short days and/or cold temperatures, and growth of terminal meristems is continuous. The *evg *trait segregates as a single recessive nuclear gene [22], and corresponds to a deletion in the linkage group one (LG1) of the *Prunus *reference genetic map [21,23]. A cluster of *SVP*-like (*SHORT VEGETATIVE PHASE*) MADS-box genes is located in this deleted region and these genes are not expressed in the peach mutant *evg *[21,24]. Three of the six *SVP*-like genes, named dormancy associated MADS-box (DAM) genes, are most likely to be responsible for the continuous growth phenotype of the mutant [25].

The *evg *mutant has been proposed as a useful system for studying winter seasonal growth behavior [22]. Recent studies have provided information regarding the putative molecular basis of the *evg *mutation as the loss of six DAM genes [21,23,26]. Here we used the *evg *mutant to investigate the development of growth arrest and endodormancy. We have used wild-type (WT) and the continuous growth, dormancy-incapable *evg *mutant genotypes to identify genes differentially expressed following transition to a SD photoperiod. Dormancy-incapable *evg *was used as a filter to reduce SD-induced differential gene expression signals common to both genotypes, and therefore not involved in signalling growth cessation and dormancy entrance. We found genes that can be placed in the photoperiod response pathway disrupted by the *evg *mutation.

## Methods

### Plant materials and growth condition

Rooted cuttings from a F_2 _sibling population segregating for WT and *evg *phenotypes were grown in Fafard 3B soilless mix (45% peat moss, 15% perlite, 15% vermiculite, 25% bark; Fafard, Agawam, MA, USA) and sand (2:1 v/v), 3.5 g L^-1 ^Osmocote 14-14-14 (Scotts, Marysville, OH, USA) and 3.5 g L^-1 ^dolomitic lime (Oldcastle, Atlanta, GA, USA), for 2 months in a greenhouse at 25°C with 16 h light/8 h dark. WT and *evg *plants were transferred to a growth room for two weeks of acclimation under long days (LD, 16 h light/8 h dark) and shifted to SD (8 h light/16 h dark) photoperiod conditions for eight weeks. In both photoperiod treatments, all other environmental conditions were identical: 250-300 μmol photon m^-2 ^s^-1 ^light intensity at canopy height was provided by AgroSun^® ^Gold 1000W sodium/halide lamps (Agrosun Inc, New York, NY, USA), temperature was 22.5°C during light and 18.7°C during dark and relative humidity was 48% during light and 55% during dark. Plants were watered every two days as needed.

Primary axis elongation was measured weekly on 29 WT and 15 *evg *plants. Re-growth potential in permissive conditions (LD) was assessed weekly following the transition to short days: replicate WT trees were transferred from SD to LD conditions and vegetative bud break and resumption of growth was observed during following two weeks.

Apical tissues were sampled from WT and *evg *trees at 0, 1, 2, 4, and 8 weeks following transfer to SD. Sixteen (WT) or eight (*evg*) apical tips were pooled from each genotype at each time for suppression subtractive hybridization (SSH). Three WT and three *evg *apical tips were harvested at each time for gene expression analysis by real-time PCR.

### RNA isolation and reverse transcription

After sampling, plant tissues were immediately frozen in liquid nitrogen and stored at -80°C. Total RNA was isolated using the protocol of Meisel et al [27]. After DNase I treatment (Invitrogen, Carlsbad, CA, USA) to eliminate possible genomic DNA contamination, 2.5 μg of total RNA were reverse transcribed using an oligo(dT)_20 _as a primer with SuperScript III first strand synthesis system for reverse transcriptase (RT)-PCR (Invitrogen).

### Suppression subtractive hybridization

Suppression subtractive hybridization (SSH) PCR between WT and *evg *samples, within each sampling date, was performed using the Clontech PCR-Select cDNA Subtraction Kit (Clontech Laboratories, Palo Alto, CA, USA), starting with 2 μg of sample polyA^+ ^RNA purified from total RNA using Dynabeads Oligo(dT)_25 _(Invitrogen). Forward-subtracted, reverse-subtracted, and unsubtracted hybridizations were performed following the manufacturer's instructions for the identification of clones enriched in one genotype relative to the other. The subtracted cDNA population of each hybridization was purified with QIAquick PCR purification kit (Qiagen, Inc., Valencia, CA, USA) and cloned in pGEM-T Easy using pGEM-T Easy cloning kit (Promega, Madison, WI, USA).

A total of 11,520 clones from subtracted cDNA libraries (1,152 per each forward and reverse subtracted library per sampling date) were screened for up-regulated or down-regulated expression in the WT or *evg *by hybridization. Clones were grown in plates, transferred to Hybond-N^+ ^filters (Amersham Biosciences, GE Healthcare Ltd, Little Chalfont Buckinghamshire, UK), lysed and DNA was fixed by oven baking. Each subtracted library was hybridized with forward- and reverse-subtracted and unsubtracted radiolabeled cDNA probes with adaptors removed to avoid the loss of low-abundance differentially expressed mRNAs. A total of 177 clones were selected as having strong hybridization signals in the selected were successfully sequenced and subjected to a BLASTx against GenBank database. Sequences without similarity were analyzed again using tBLASTx or BLASTn. Sequences were evaluated for redundancy, and differential expression between WT and *evg *was confirmed by real-time PCR.

### Expression analysis by real-time PCR

Real-time PCR was performed on an iCycler iQ system (Bio-Rad, Hercules, CA, USA) using the iQ SYBR-Green Supermix (Bio-Rad, Hercules, CA, USA). Gene-specific primers for each of the selected genes were used (Table 1) to amplify products from synthesized cDNA samples with the SuperScript III first strand synthesis system for reverse transcription (RT)-PCR (Invitrogen). Three technical replications for each of the three biological replicates were performed. PCR was conducted with the following program: an initial DNA polymerase activation at 95°C for 180 s, then followed by 40 cycles of 95°C for 30 s, 60°C for 30 s, and 72°C for 30 s. Finally, a melting curve was performed, and the PCR products were checked with 2% agarose gel in 1× TAE with ethidium bromide.

**Table 1:** Gene bank accession numbers and primer sequences used in real-time PCR of differentially expressed ESTs

Gene name	EST bank accession #		Forward (F) and reverse (R) primer sequence
Desiccation-related protein, putative	GE653173	F	5'-AGGGCTCGACGATATCAGTCC-3'
		R	5'-TGCATACGGGTCAAATGCAGG-3'
Amidase family protein	GE653170	F	5'-CGGTTCGATCCTTGGCAGAC-3'
		R	5'-TCGACACGGCTGCAAATGGAG-3'
Deoxynucleoside kinase family protein	GE653171	F	5'-AGGAGGACAGCTCGAACTCAG-3'
		R	5'-GCATACCTCTGCGGCTCAGC-3'
Auxin-binding protein ABP20 precursor	GE653207	F	5'-AGCCTCACCTCCATTGACTTGG-3'
		R	5'-TGTTGCTCAGTTTCCTGGTGTGA-3'
Amino acid transporter family protein	GE653321	F	5'-GGCTTCACCCATGACATCACC-3'
		R	5'-CTGGAATTATGAGCCTGCCTGC-3'
Glycoside hydrolase, family 18	GE653328	F	5'-CAGTCCACCACTCCCATCACTG-3'
		R	5'-GCTTCCATTGCTCCCTTCGATG-3'
ATP sulfurylase 1	GE653245	F	5'-ACAAGACGCAATGCTGATGCTG-3'
		R	5'-ACCACAGTCGTCTCAGGATCAAG-3'
KEG (KEEP ON GOING) protein	GE653332	F	5'-ACCCGTTCTATTTCCGATGCCT-3'
		R	5'-TCAGTTTCAACTCCAACCCACCA-3'
Phosphatidylinositol 3- and 4-kinase family protein	GE653311	F	5'-GGGTTGGGGAGACAGGTTTCA-3'
		R	5'-AGTCCCATGATCACTGGCATCA-3'
PRH75 (DEAD-box helicase)	GE653257	F	5'-TGTAGCCAGCAGCCTTAGCAAG-3'
		R	5'-GCCAGTTGATGTTGCCAAAGCAG-3'
Zinc ion binding/LIM	GE653319	F	5'-AGAAGAGGATGAGGAGGAAGACG-3'
		R	5'-GTTGTTGACAGGGTCGATTCTGG-3'
ATP-binding cassette transporter MRP6	GE653330	F	5'-CTGGGATTGTGGGTAGAACTGG-3'
		R	5'-CCTTCAAACATGGTTGGGTCCTG-3'
Unknown1	GE653303	F	5'-CTCTCTGTGCTTCTCTCTCCTCA-3'
		R	5'-TCCAGATTAACTCAGGGAGAAACCAG-3'
Late embryogenesis abundant (LEA)	GE653244	F	5'-TTCAAATTCTCCGGGGGTCG-3'
		R	5'-TTCCAGGCCATCTTCCACGG-3'
Metallothionein-like protein	GE653329	F	5'-TCCACCAATCAACAAACACCTCAC-3'
		R	5'-TAGCAAGTAATCTATGCGTGTGTGG-3'
Pathogenesis-related protein 1a (PR-1a)	GE653248	F	5'-CGACTGCAATCTTGTCCACTCTGG-3'
		R	5'-ACCTCCACTGTTGCACCTCAC-3'
Dormancy associated MADS-box gene 1 (PpDAM1)	GE653327	F	5'-CAGAGGGCAAGCAACTACCAC-3'
		R	5'-CCAGAGAAATTATGGAAGCCCCA-3'
Dormancy associated MADS-box gene 6 (PpDAM6)	GE653238	F	5'-CCAACAACCAGTTAAGGCAGAAGA-3'
		R	5'-GGAAGCCCCAGTTTGAGAGA-3'
Epicotyl-specific tissue protein	GE653203	F	5'-CACCAAAAGAGAAAGCCGACTGC-3'
		R	5'-TCAACCTCAACGTCAACCTCAAC-3'
RD22 (dehydration-responsive) precursor	GE653312	F	5'-GAACCCACACAAGATTATCAGCAGG-3'
		R	5'-TTCTACTGCCACAGCCAGCA-3'
Unknown2	GE653334	F	5'-ATGGCAAACCACCAAGCACTCA-3'
		R	5'-GTAGAGGAGCCTTGATTGGAGGAG-3'
Unknown3	GE653309	F	5'-AAGTTGTCCATCCCAACACATTCG-3'
		R	5'-GCGAGGACATCTCTGGCAATAAGA-3'
Unknown4	GE653307	F	5'-TCCTCAACGAACAGACGGAACTC-3'
		R	5'-TGGTGGTCTTGGAAATGCTGGT-3'

Fluorescence values were baseline-corrected and averaged efficiencies for each gene and Ct values were calculated using LinRegPCR program [28]. Gene expression measurements were determined with the Gene Expression Ct Difference (GED) formula [29]. The gene expression levels were normalized to a peach EST (GenBank Accession Number DY652828), similar to the *Arabidopsis thaliana *expressed gene At5g12240 [30], and were expressed relative to the values at week 0 (LD). The reference gene At5g12240 showed a low variability of expression within biological replicates and a stable expression throughout the experiment a with a stability index of 0.12 for WT and 0.25 for *evg *(calculated as in [31]). The reference gene At5g12240 showed better stability index values than α-tubulin (from EST Tua5, GenBank Accession DY650410).

### Statistical analysis

Statistical testing of quantitative expression level between WT and *evg *within sampling date was performed with the Mann-Whitney-Wilcoxon test (*P *< 0.05). Growth elongation was analyzed with the two-sample paired t-test (*P *< 0.05) at each sampling date. Analyses were performed using the statistical software version package of SAS v.9.1.3 (SAS Institute Inc., Cary, NC).

## Results

### Short days rapidly induce growth cessation in WT plants

**Figure 1:**
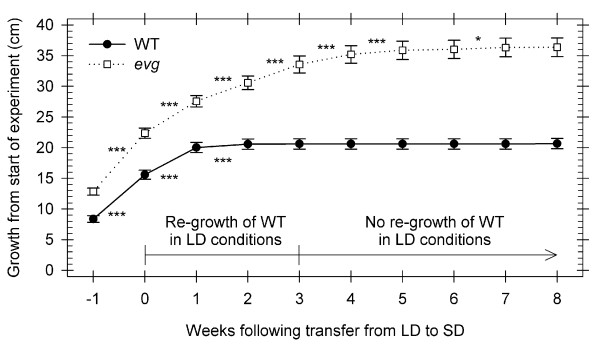
**Stem growth and potential re-growth of WT and *evg *plants after transferring to SD**. Stem growth (increase in length) was measured one week prior and eight weeks after transfer of WT and *evg *plants from LD (16 h light) to SD (8 h light) photoperiod conditions and potential re-growth when WT plants are transferred back to LD conditions. The day of treatment change to SD conditions is named week 0. Data are mean ± SE of 29 and 15 replicates for WT and *evg*, respectively. Growth elongation significance between paired dates is indicated: not significant, not showed; *, *P *< 0.05, ***, *P *< 0.001.

WT plants showed apical growth cessation after two weeks of SD (Figure 1). Plants were unable to resume growth in LDs after three weeks of SD exposure (Figure 1). *Evg *plants did not show slowed growth until several weeks of SD exposure (Figure 1), but were able to immediately resume growth when transferred to LDs even after eight weeks of SD treatment. The slowed growth observed in the *evg *plants at the end of the experiment was likely caused by the decreased total integrated light exposure resulting from reducing the light period from 16 to 8 hours without altering the light intensity.

### Differentially expressed genes and functional classification

cDNAs prepared from WT and *evg *apical tissue were used as testers and drivers for SSH PCR. A total of 11,520 clones from subtracted cDNA libraries were screened for up-regulated or down-regulated expression in the WT or *evg *by hybridization. After comparing the signal among forward- and reverse-subtracted and unsubtracted radiolabeled cDNA probes, we selected 177 clones for sequencing. These 177 sequences assembled into 106 contigs. The majority of the 106 sequences were up-regulated in WT relative to *evg*. Following the selection of the highest signal spots and verification by real-time PCR, we identified 23 genes as up-regulated in the WT relative to *evg *in response to SD conditions.

Differentially expressed genes were assigned to five categories according to their putative functions generated by similarity searches against the GenBank database. Although the number of genes obtained was very limited, the largest group of genes (30%) was signaling/transcription related genes followed by genes with unknown (26%) and defense functions (22%). One of the unclassified proteins (unknown3) had no sequence similarity in GenBank.

**Figure 2:**
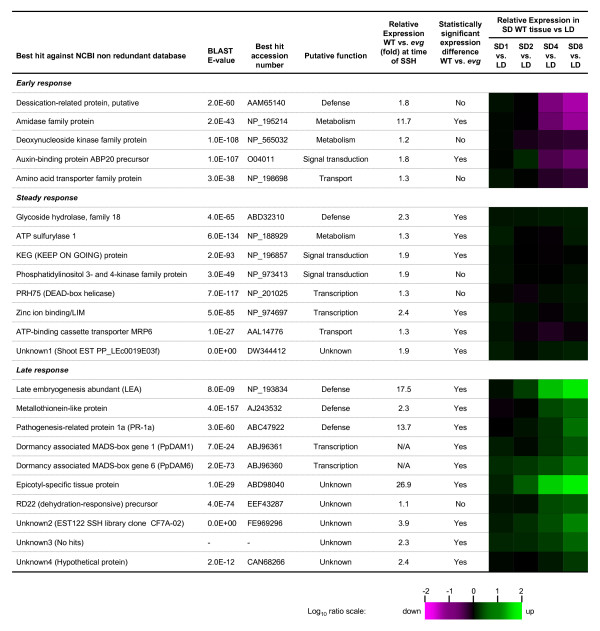
**Putative differentially expressed genes between WT and *evg *and their expression pattern during SD in WT**. Sequences were analyzed using BLASTx tool and using tBLASTx or BLASTn when no similarity was found. Statistical testing of expression level between WT and *evg *was performed with the Mann-Whitney-Wilcoxon test (*P *< 0.05). Gene expression pattern in WT tissue was calculated as the expression values in SD relative to the values at LD conditions. The color scale is in log_10 _ratio where a green color corresponds with up-regulated gene in SD, the magenta color with down-regulated color in SD and black color with no change in the expression level.

Seventeen of the 23 genes showed statistically significant increased expression with real-time PCR in the WT relative to *evg *(Figure 2) by ANOVA with the Mann-Whitney test. The expression of two DAM genes, PpDAM1 and PpDAM6, was observed in WT tissue and as expected we did not detect expression of these genes in *evg *tissue by real-time PCR.

### Gene expression in SD conditions

**Figure 3:**
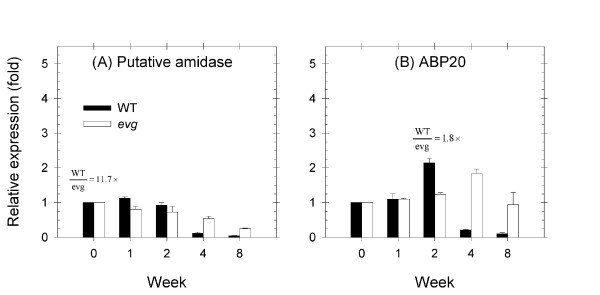
**Expression profiles of early response genes in WT and *evg *apical tissue**. Putative amidase (A) and auxin-binding protein ABP20 precursor (B) gene expression is shown relative to the LD level (week 0 prior to the change in photoperiod) for each genotype. Values above columns represent the relative expression (fold) between WT and *evg *apical tissues at the week where it was maximum.

We measured the expression response of the 23 genes identified above to the LD to SD transition in WT tissues by real-time PCR. Gene expression in the WT following the LD to SD transition showed three distinct patterns (Figure 2). The first group of genes had a stable or increased expression immediately following transition to SD peaking at two weeks. The expression peak of these genes coincides with growth cessation in the WT. After two weeks in SD, expression of these genes then decreased to values similar or below those in LD conditions (Figure 2). Defence, metabolism, signalling/transcription and transport genes were included in this group. The putative amidase showed stable expression in both WT and *evg *plants in the first and second weeks after transfer to SD, followed by down-regulation in both genotypes, although its expression decreased faster in the WT compared with *evg *(Figure 3A). The auxin-binding protein 20 (ABP20) transcript showed a transient up-regulation after the second week of SD followed by down-regulation in WT apical tissue, whereas the expression remained stable in *evg *(Figure 3B).

**Figure 4:**
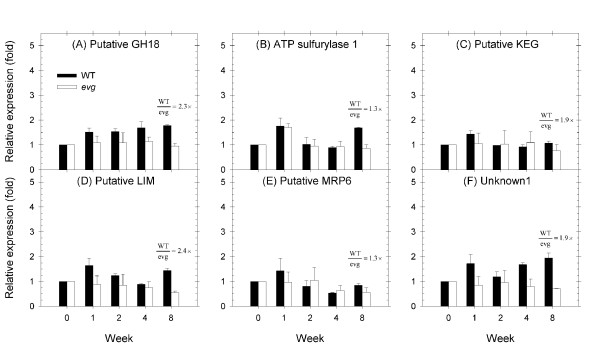
**Expression profiles of steady response genes in WT and *evg *apical tissue**. Putative GH18 (A), putative ATP sulfurylase 1 (B), putative KEG (C), putative LIM (D), putativeMRP6 (E) and unknown1 (F) gene expression is shown relative to the LD level (week 0 prior to the change in photoperiod) for each genotype. Values above columns represent the relative expression (fold) between WT and *evg *apical tissues at the week where it was maximum.

The second group of genes had an increased expression in WT tissue immediately following transition to SD that was maintained steady until the end of the experiment or similar to the LD values (Figure 2). The putative glycoside hydrolase 18 (GH18), ATP sulfurylase 1, KEG (KEEP ON GOING), zinc ion binding/LIM, ATP-binding cassette transporter MRP6 and unknown1 followed this profile in WT (Figure 4A-F). However, in general the expression of these genes remained stable in *evg*.

**Figure 5:**
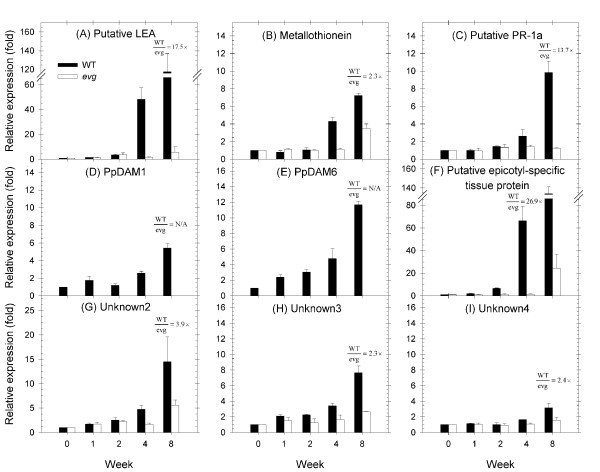
**Expression profiles of late response genes in WT and *evg *apical tissue**. Putative LEA protein (A), metallothionein (B), PR-1a (C), PpDAM1 (D), PpDAM6 (E), putative epicotyl-specific tissue protein (F), unknown2 (G), unknown3 (H) and unknown4 (I) gene expression is shown relative to the LD level (week 0 prior to the change in photoperiod) for each genotype. Values above columns represent the relative expression (fold) between WT and *evg *apical tissues at the week where it was maximum.

The third group of genes had a delayed response in WT tissue. In general, their expression increased after one to two weeks of SD exposure and continued to increase until the end of the experiment (Figure 2). Defence, unknown, and signalling/transcription genes were included in this second group. The putative late embryogenesis abundant (LEA) protein, metallothionein, pathogenesis-related protein 1a (PR-1a), PpDAM1, PpDAM6, epicotyl-specific tissue protein, unknown2, unknown3 and unknown4 genes followed this profile in WT (Figure 5A-I). However, in general the expression of these genes remained stable in *evg*. Two of these genes, the putative LEA and epicotyl-specific tissue protein genes, showed a large up-regulation the last week of the experiment. The expression of putative LEA and epicotyl-specific tissue protein genes in WT was 118- and 134-fold up-regulated, respectively, eight weeks after transferring to SD relative to LD, whereas their expression in *evg *was only up-regulated 6- and 24- fold, respectively (Figure 5A, F).

## Discussion

Understanding of the regulatory network involved in vegetative growth cessation and dormancy induction is still limited [1,2,32]. We used SSH PCR to identify differentially expressed genes in apical tissue between WT peach and the dormancy-incapable *evg *mutant.

We found 17 significantly up-regulated genes in the WT with respect to the mutant. Interestingly, more than 25% of the genes could not have a putative function assigned. A similar proportion of unclassifiable genes were reported in previous studies of dormancy in woody species indicating that representation of seasonally expressed genes in existing databases is low [14].

When considering the WT expression changes following transfer from LD to SD, three patterns could be defined. A first group of genes showed expression only during two weeks after transfer to SD. A second group of genes showed increased expression since the first week after transfer to SD and that was maintained steady or then was similar to values before the transfer. The third group of genes showed progressively enhanced expression throughout all weeks, and especially following growth cessation (weeks 4 to 8). Two major phases of gene expression response to SD were found previously in poplar: an early response to SD during the first two weeks and then a late adaptation [5]. In another study in poplar, gene expression changed after about three weeks of SD, when bud scales were visible, and after this point there was a large reduction in the number of expressed genes and their expression level [7].

An interesting case of early response is the ABP20 gene whose expression peaked coincident with growth cessation and decreased 10-fold after terminal meristems were unable to resume growth (weeks 4 and 8). The peach ABP20 is related to germin and germin-like genes, which belong to the ancient superfamily of cupin proteins. The ABP20 contains a region which shared 40% of amino acid identity with a putative auxin binding site in ABP1, an auxin-binding protein isolated from maize coleoptiles [33]. This region of homology corresponds with a BoxA domain, whose structure has been suggested to be conserved among proteins that have auxin binding-activity [33,34]. The localization of ABP20 in the cell wall and its ability to produce H_2_O_2 _suggest a similar biological function to germin, which is related with expansion and lignification of the cell wall [35]. The ABP1 protein of *Arabidopsis *has been also associated with the auxin-induced cell elongation [36] and has been found to be essential for the auxin control of the cell cycle using tobacco cell culture [37]. Recent studies support the hypothesis of an auxin extracellular receptor role for ABP1 [38,39]. ABP20 gene expression throughout the development of peach vegetative buds was previously reported [35]. In a recent proteomic analysis, the ABP20 protein content in peach bark tissue decreased in after 5 weeks of SD treatment [40]. Several genes involved in auxin metabolism and transport were found down-regulated in the same tissue type and conditions [15]. It has been observed that auxin levels do not change in cambial cells during the dormancy period, but the responsiveness to auxin does [1,41]. Although not definitive, it is tempting to speculate that there may be a role for ABP20 protein in the process of growth cessation in bud tissue by modulating the perception of auxin. However, this hypothesis will have to be specifically tested.

Another early responding gene is the putative amidase. Differential expression of the amidase gene could correspond with the different rate of growth between WT and *evg *genotypes, due to the core metabolic function of amidase proteins, however, a specific signalling role cannot be dismissed.

The putative LIM and KEG genes are two cases of steady response with up-regulation during the first week after transfer to SD with this elevated expression maintained similar after that point. Functional analysis is lacking for the peach putative LIM. The LIM protein gene family participates in processes such as gene transcription, cellular organization and signalling [42]. Their essential roles have been well characterized in animals; however, only a few members have been studied in plants [42]. A better characterized protein is KEG, a protein capable of mediating ubiquitylation. In Arabidopsis, KEG has an essential role in ABA signalling. During post-germination development, KEG protein is found in *Arabidopsis *seedlings [43]. The model proposed for KEG function is the ubiquitylation and subsequent degradation of ABI5 (ABSCISIC ACID-INSENSITIVE5) and ABI3 by KEG in the absence of ABA, thus decreasing their ability to suppress growth. In the presence of ABA, this degradation is slowed to allow the transduction cascades resulting in a suppression of growth [43]. There are commonalities between bud and seed dormancy, and although the inducing mechanism might not be shared directly, similar signalling circuits could be adopted [1].

Other steady responding genes are the putative GH18 family gene and unknown1. The GH18 subfamily includes chitinases with diverse defence-related functions. Some of them do not have chitinase activity [44], although the putative glycoside hydrolase found in this work exhibited a conserved motif that dictates enzymatic activity. Its expression was found to be up-regulated in WT. GH18 transcripts were found preferentially in active rather than dormant poplar buds [45]. Several chitinases associated with defence-related functions have been found to be up-regulated in *Populus *dormant cambium tissue and peach bark tissue during dormancy induction [6,15]. The unknown1 sequence showed similarity to shoot and fruit peach ESTs, but this is the first report of the regulation of this gene.

During the late response, there is a large up-regulation of the defence-related genes LEA, metallothionein and PR-1. LEA proteins have the presumed role of cellular stabilizers under stress conditions. An *Arabidopsis *LEA domain-containing gene (At4g21020) similar to the peach gene reported here was found expressed in seeds of *Arabidopsis *[46]. The increase in LEA expression can be related to the cold acclimation induced by photoperiod, as a protective measure against dehydration. This adaptation to dehydration was also previously found starting in the first weeks of SD-dormancy induced in poplar [5,7]. In contrast, a previous study found that SD induced a down-regulation of a different LEA protein in peach bark [15]. LEA genes have been found down-regulated during the dormancy release in raspberry [13] and oak buds [9]. If the LEA gene we have identified is indeed involved in dehydration resistance or cold hardiness, the lagging LEA expression we observed in the *evg *mutant is consistent with the impaired cold hardiness response previously observed in seasonal LEA expression in *evg *and deciduous genotypes of peach [47].

Putative metallothioneins were found up-regulated during dormancy release in raspberry [13] and Norway spruce [8], whereas other metallothioneins were found up-regulated during dormancy development in poplar buds [4], in dormant cambial tissue in aspen [6] and during chilling accumulation in grape [14]. Similar metallothioneins to the peach sequence found in our experiment were also expressed during fruit development in apricot and in response to cold stress in apple fruit [48]. Several roles have been defined for metallothioneins: detoxification of heavy metals, homeostasis of essential metal ions, and regulation of gene expression in development processes.

The class 1 pathogenesis-related proteins are not only involved in plant defence responses, but also in development [49]. However, little is known about the molecular function of class 1 pathogenesis-related proteins in plant signalling networks during development. A dual function for some pathogenesis-related proteins as antifreeze proteins during dormancy has been proposed [40]. An increase in PR-1 expression was similarly found during dormancy entrance in poplar [4].

The non-dormant phenotype of the peach *evg *corresponds to a deletion in the LG1 group of the general genetic map [21,23]. A cluster of DAM genes that belong to the *SVP*-subfamily of MADS-box genes are located in this deleted region [24]. Three of these genes, PpDAM1, PpDAM2 and PpDAM4 are the most likely candidates for the regulation of growth cessation and terminal bud formation [25]. In this work, two of the DAM genes, the PpDAM1 and PpDAM6, were detected and differentially expressed between WT and *evg*. Their expression was up-regulated after the change in photoperiod and increased continually during bud development. A *SVP*-like MADS-box factor similar to the PpDAM6 gene showed endodormancy-associated expression in lateral buds of Japanese apricot [16]. Additionally, two putative SVP-like genes, with sequences similar to the PpDAM6 and PpDAM1 genes, were down-regulated during the dormancy release in *Rubus idaeus *L. buds [13]. The PpDAM6 gene is induced by short photoperiods [25] and unpublished data from our lab shows it to be cold-suppressed. There are six peach DAM genes expressed in WT trees and all six are not expressed in the mutant *evg *[25]. Here only two of the six genes we know should be definitely differentially expressed between the WT and mutant were detected with the SSH PCR technique we used in this study. This is in line with the known limited sensitivity of SSH for isolating genes like transcription factors that are expressed at low absolute levels.

The most strongly up-regulated gene after several weeks of SD photoperiod inducing-conditions was similar to the epicotyl-specific tissue protein from *Striga asiatica*. A similar protein in *Cicer aeretinum*, CanST-2, seems to have an opposite expression pattern, since its transcript level decrease when the growth of epicotyls is inhibited [50]. However, the molecular function of the epicotyl-specific tissue protein in the bud development process remains unknown. A similar protein was found to be down-regulated by low temperatures in peach bark [15].

Three additional genes of unknown function were found up-regulated after several weeks of SD photoperiod. Unknown2 expression was induced by SD photoperiod and cold in other study of SD responses in peach [15]. The unknown4 sequence showed similarity to a hypothetical protein of *Vitis vinifera*; however, a putative function and relationship with growth cessation or dormancy could not be assigned. The unknown3 sequence represented a novel transcript in plants. These unknown genes can now be associated with SD responsiveness in peach and may represent novel components of growth cessation and/or dormancy development in peach or other perennial species. Release of the assembled peach genome sequence (ongoing, Dr. Doreen Main, personal communication) will allow the localization of these genes in the genome and determining if they co-localize with genetic and physical map locations known to regulate phenological events such as bud set, chilling requirement, or bud break [51].

## Conclusions

The use of the mutant that fails to undergo growth cessation *evg *as a biological filter in controlled conditions has allowed us to reduce the number of genes detected by typical differential display experiments during growth cessation and dormancy. The identified genes are putatively involved in growth cessation and/or dormancy entrance and should be downstream of *EVG *in this pathway. Future proteomic and physiological experiments are required to verify their role in growth cessation and/or dormancy establishment.

## Authors' contributions

SJ and LZ carried out the SSH experiment and drafted the manuscript. SJ carried out the real-time PCR analyses. GLR assisted in the analysis of the results and drafting of the manuscript. DBG conceived of the study, participated in its design and assisted in the drafting of the manuscript. All the authors read and approved the final manuscript.
